# Asymmetric right/left encoding of emotions in the human subthalamic nucleus

**DOI:** 10.3389/fnsys.2013.00069

**Published:** 2013-10-29

**Authors:** Renana Eitan, Reuben R. Shamir, Eduard Linetsky, Ovadya Rosenbluh, Shay Moshel, Tamir Ben-Hur, Hagai Bergman, Zvi Israel

**Affiliations:** ^1^Department of Psychiatry, Hadassah-Hebrew University Medical CenterJerusalem, Israel; ^2^Department of Medical Neurobiology (Physiology), Institute of Medical Research – Israel-Canada, The Hebrew University-Hadassah Medical SchoolJerusalem, Israel; ^3^The Edmond and Lily Safra Center for Brain Research, The Hebrew UniversityJerusalem, Israel; ^4^Department of Neurosurgery, Center for Functional and Restorative Neurosurgery, Hadassah-Hebrew University Medical CenterJerusalem, Israel; ^5^Department of Neurology, Hadassah–Hebrew University Medical CenterJerusalem, Israel; ^6^The Jerusalem Mental Health Center, Kfar-Shaul EtanimJerusalem, Israel

**Keywords:** Parkinson's disease, deep brain stimulation (DBS), emotions, subthalamic nucleus, spikes

## Abstract

Emotional processing is lateralized to the non-dominant brain hemisphere. However, there is no clear spatial model for lateralization of emotional domains in the basal ganglia. The subthalamic nucleus (STN), an input structure in the basal ganglia network, plays a major role in the pathophysiology of Parkinson's disease (PD). This role is probably not limited only to the motor deficits of PD, but may also span the emotional and cognitive deficits commonly observed in PD patients. Beta oscillations (12–30 Hz), the electrophysiological signature of PD, are restricted to the dorsolateral part of the STN that corresponds to the anatomically defined sensorimotor STN. The more medial, more anterior and more ventral parts of the STN are thought to correspond to the anatomically defined limbic and associative territories of the STN. Surprisingly, little is known about the electrophysiological properties of the non-motor domains of the STN, nor about electrophysiological differences between right and left STNs. In this study, microelectrodes were utilized to record the STN spontaneous spiking activity and responses to vocal non-verbal emotional stimuli during deep brain stimulation (DBS) surgeries in human PD patients. The oscillation properties of the STN neurons were used to map the dorsal oscillatory and the ventral non-oscillatory regions of the STN. Emotive auditory stimulation evoked activity in the ventral non-oscillatory region of the right STN. These responses were not observed in the left ventral STN or in the dorsal regions of either the right or left STN. Therefore, our results suggest that the ventral non-oscillatory regions are asymmetrically associated with non-motor functions, with the right ventral STN associated with emotional processing. These results suggest that DBS of the right ventral STN may be associated with beneficial or adverse emotional effects observed in PD patients and may relieve mental symptoms in other neurological and psychiatric diseases.

## Introduction

Subthalamic nucleus (STN) deep brain stimulation (DBS) is an established therapy for the management of motor symptoms of advanced Parkinson's disease (PD; Benazzouz et al., 1993; Benabid et al., 1994; Weaver et al., 2009; Follett et al., 2010; Moro et al., 2010; Bronstein et al., 2011; Lhommée et al., 2012; Odekerken et al., 2013; Schuepbach et al., 2013), and is also a promising potential therapy for the management of obsessive-compulsive disorder (OCD) (Mallet et al., 2008; Chabardès et al., 2012).

Psychiatric adverse effects such as apathy, depression, emotion recognition and reactivity, hypomania, and suicide have been observed in PD patients before and after STN DBS (Dujardin et al., 2004; Schroeder et al., 2004; Biseul et al., 2005; Temel et al., 2006; Drapier et al., 2008; Witt et al., 2008; Péron et al., 2010a). Castner et al. (2007) have observed delayed reaction times for negative valence words in healthy control volunteers and for PD patients “on” stimulation, but not PD patients in the “off” stimulation condition. In addition, recognition of negative emotions (fear, anger, and sadness) expressed visually (facial expressions) or vocally was significantly impaired in PD patients after STN DBS (Péron et al., 2010a,b). PD patients “on” stimulation reported significantly less intense feelings of fear, anxiety, and disgust for film excerpts intended to induce “fear” as compared with the pre-operative and the control groups (Vicente et al., 2009).

In contrast, overall *improvement* in neuropsychiatric symptoms in PD patients following STN DBS has recently been reported (Lhommée et al., 2012). STN DBS of PD patients in the “off” medication state was associated with a reduction in the frequency and severity of non-motor fluctuations (Ortega-Cubero et al., 2013), and reduce compulsive use of dopaminergic medication and its behavioral consequences (Eusebio et al., 2013). Thus, the STN is not a pure motor structure, but also involved in emotional processing (Péron et al., 2012).

Although there is considerable anatomical evidence that supports the segregation of the STN into distinct limbic, associative and motor zones (Lambert et al., 2012; Haynes and Haber, 2013), there is no widely accepted spatial model for the physiological correlates of this subdivision (Brunenberg et al., 2012; Buot et al., 2012). Emotional processing is considered to be lateralized and attributed to the non-dominant hemisphere (Gainotti, 2012). Right/left asymmetry of dopamine deficits may differentially impact emotion and cognition in PD patients (Tomer and Aharon-Peretz, 2004; Lambert et al., 2012; Ventura et al., 2012). Piallat et al. (2011) have reported that burst neurons were predominantly left-sided in the STN of OCD but bilateral in PD patients. Such right/left asymmetry has not been observed in the STN of PD patients either by means of imaging modalities or macro-electrode local field potential (LFP) recordings (Kühn et al., 2005; Buot et al., 2012; Lambert et al., 2012).

To better understand the limbic role of the STN, micro-electrode unit activity in PD patients undergoing STN DBS was recorded and analyzed during intraoperative vocal emotional stimulation. This study utilized the oscillatory activity that characterizes the PD STN for segmentation of motor and non-motor regions. STN oscillatory activity in the beta (12–30 Hz) band is associated with akinetic-rigid PD symptoms and is observed in the dorso-lateral oscillatory region (DLOR) of the STN that corresponds to the anatomically defined sensory-motor region (Zaidel et al., 2010). Beta band oscillations are less likely to be observed at the ventro-medial non-oscillatory (VMNR) STN region that is probably related to cognitive and limbic functions. This study tested whether the electro-physiological response to an emotional stimulus would be observed in the ventro-medial part of the STN, and furthermore whether it would be lateralized to the non-dominant hemisphere in similarity to the cortex.

## Methods

Clinical characteristics of PD patients (*n* = 17) of this study are given in Table 1. All patients met accepted inclusion criteria for DBS surgery and signed informed consent. This study was authorized and supervised by the IRB of Hadassah Medical Center (reference code: 0168-10-HMO).

**Table 1:** **Clinical characteristics of patients with Parkinson's disease at time of surgery**.

**Patient no**	**Sex**	**Age (years)**	**Disease duration (years)**	**Dominant hand**	**Surgery side**	**ACE**	**FAB**	**HAM-21**	**UPDRS OFF (total score)**	**UPDRS OFF (I; II; III; IV)**
1	M	55	10	R	Unilateral, left	90	16	10	110	1;26;75;8
2	M	70	8	R	Unilateral, left	91	17	32	86	3;18;55;10
3	M	63	30	R	Staged bilateral^**^	80	14	19	90	0;15;65;10
4	F	49	14	R	Bilateral	95	14	15	85	0;14;59;12
5	M	61	8	R	Bilateral^*^	94	14	8	48	0;13;32;3
6	F	66	3.5	R	Staged bilateral	91	18	7	68	0;20;46;2
7	M	71	22	R	Staged bilateral^**^	79	11	6	93	0;32;51;10
8	M	73	15	R	Staged bilateral	88	17	16	93	2;26;57;8
9	M	65	4	R	Staged bilateral	88	18	16	81	1;20;56;4
10	F	54	7	R	Bilateral^**^	98	18	18	84	0;20;56;8
11	M	53	3	R	Bilateral	87	17	9	91	2;25;49;15
12	M	65	20	R	Bilateral	92	16	11	58	0;18;35;10
13	M	51	20	R	Bilateral	93	17	11	82	3;21;53;5
14	M	65	8	R	Bilateral	95	17	7	69	0;18;48;5
15	M	66	5	R	Unilateral, left	89	17	2	84	1;22;55;6
16	F	68	15	R	Unilateral, left	83	11	8	60	1;17;35;8
17	M	60	8	R	Unilateral, left	75	12	12	82	3;25;48;6
Sum/average ± SD	M: 13 F: 4	62.1 + 7.3	11.8 ± 7.6	R/L hand dominant-17 /0	Bilateral: 12 Unilateral, left: 5	88.7 ± 6.2	15.5 ± 2.4	12.2 ± 6.9	79.9 ± 15.2	1.0 ± 0.85; 20.6 ± 4.9; 51.4 ± 10.8; 7.6 ± 3.3

*,** Right/Left trajectory was excluded for artifacts and noise; ACE-R, Addenbrooke's Cognitive Examination - Revised (range 0–100, lower scores indicate cognitive decline); FAB, Frontal Assessment Battery (range 0–18, lower scores indicate cognitive decline); HAM-21, Hamilton Depression Scale (range 0–67, higher scores indicate depressed mood); UPDRS OFF, Unified Parkinson's Disease Rating Scale off medication (range 0–199, higher scores indicate advanced dysfunction here and in parts I-IV); UPDRS Part I: clinician-scored mentation, behavior, and mood evaluation (range 0–16); UPDRS Part II: clinician-scored activities of daily life evaluation (range 0–52); UPDRS Part III: clinician-scored motor evaluation (range 0–108); UPDRS Part IV: clinician-scored evaluation of complications of therapy (range 0–23).

STN evoked responses to emotional vocal stimulus were analyzed. To avoid a bias, the emotional stimulus should incorporate a variety of emotions and be culture and language independent as much as possible. For this purpose, the Montreal affective voices (MAV) database was selected and validated. The MAV consists of negative, neutral and positive non-verbal male and female voices (Belin et al., 2008). The emotional voices were played to the PD patients during DBS surgery synchronized with microelectrode recordings of the STN. The data was then analyzed to define the areas of the STN at which the emotive vocal stimulation resulted in a modulation of the neuronal activity. In the following sections the above paradigm is described in more detail.

### Validation of emotional voices database

The Montreal affective voices, a validated tool for research on auditory affective processing (Belin et al., 2008) was used in this study. During vocal communication, listeners attend to speech prosody to infer the emotions or the affective mood of the speaker. Non-verbal affective processing based on auditory recognition does not contain verbal context and is therefore valid across different countries and cultures. Moreover, it is probably less dependent on brain areas that are specialized for language processing, and therefore reflects a purer emotional activity. To validate the Montreal affective voices for Hebrew speakers, the computerized Montreal affective voices question battery was translated to Hebrew and validated with 29 healthy volunteers (>60 years, 66.7 ± 5.2 years, mean ± SD, *n* = 14, 8 females and 6 males; and <60, 34.9 ± 8.9 years, mean ± SD, *n* = 15, 8 females and 7 males). Whilst on their regular dopamine replacement therapy, the emotive voices were played to all patients (*n* = 17) the day before surgery such that they would become more familiar with the MAV in the operating room. Thirteen patients (62.3 ± 7.5 years old, mean ± SD, three females and 10 males) also answered the Montreal affective voices battery that assesses subjective information regarding the valence and arousal of the played voices.

For unbiased comparison of the PD patients and healthy volunteers, an age and gender matched group of 12 healthy volunteers was selected (60.5 ± 13.1 years old, mean ± SD, three females and nine males; shuffling of the members of this group have resulted in similar results). To estimate the accuracy of the recognition of emotive voices (recognition accuracy) the valence of the emotional voices were considered to be 100, 0 and −100 for positive, neutral, and negative voices, respectively. The patients and volunteers were asked to rank the valence on a continuous scale from −100 (negative) to 100 (positive). The recognition accuracy was defined as the absolute difference between the database values and the patient evaluation.

### Surgery and microelectrode recording

Surgery, recording and data analysis methods used to evaluate the response to the emotive stimuli and to discriminate between dorsal and ventral regions of the STN are similar to those reported in our previous study (Shamir et al., 2012). Briefly, surgery was performed using the CRW stereotactic frame (Radionics, Burlington, MA, USA). STN target coordinates were chosen as a composite of indirect targeting based on the anterior commissure—posterior commissure atlas based location and direct targeting with three Tesla T2 magnetic resonance imaging (MRI), using Framelink 5 software (Medtronic, Minneapolis, USA). All recordings used in this study were made while the patients were awake and not under sedation. The patient's level of awareness was continuously assessed clinically, and if drowsy the patient was stimulated and awoken through conversation by a member of the surgical team. The side (right/left) of the first trajectory was chosen according to the severity of the Parkinsonian symptoms (right/left side first—7/10 patients respectively). The DBS procedures were done off dopaminergic medications (>12 h since last medication).

Microelectrode recording (MER) data was acquired with the MicroGuide system (AlphaOmega Engineering, Nazareth, Israel). Neurophysiological activity was recorded via polyamide coated tungsten microelectrodes (Alpha Omega) with impedance: 0.59 ± 0.13 MΩ (mean ± SD, measured at 1 kHz at the beginning of each trajectory). The signal was amplified by 10,000, band-passed from 250 to 6000 Hz, using a hardware four-pole Butterworth filter, and sampled at 48 kHz by a 12-bit A/D converter (using ±5 V input range). LFPs were not recorded due to constraints of electrical noise in the operating room. For both the left and right hemispheres, a microelectrode-recording trajectory using two parallel microelectrodes was made, starting at 10 mm above the calculated target (center of the STN trajectory as per imaging). A “central” electrode was directed at the center of the dorsolateral STN target, and an “anterior” electrode was advanced in parallel, 2 mm anterior and ventral to the central electrode. A typical trajectory was ~60° from the axial anterior commissure–posterior commissure plane and ~15° from the mid-sagittal plane. Final trajectory plans were slightly modified to avoid the cortical sulci, the ventricles and major blood vessels.

**Figure 1:**
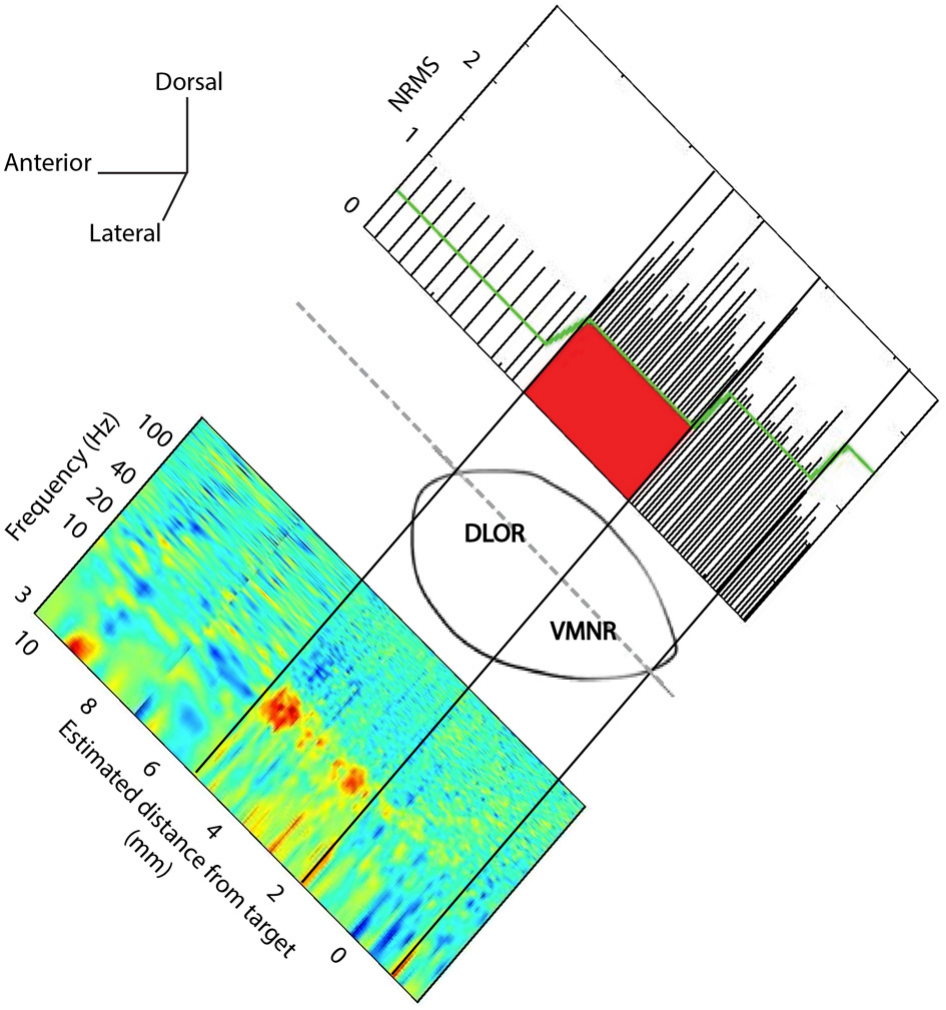
**STN trajectory and analysis of microelectrode recordings (MER)**. The parasagittal plane of the STN (atlas of Schaltenbrand and Wahren, 1977) is represented at laterality of 12 mm with respect to the AC-PC line. Normalized root mean square (NRMS) was computed on the MER to delineate the STN boundaries (upper image). The *x*-axis is the estimated distance of MER from the STN target as defined on the pre-operative MRI image. Power spectral density was computed at each MER site, and a spectrogram visualizing the change of oscillatory activity with location before and within the STN is presented (lower image).

Spontaneous and evoked STN multi-unit MER activity was recorded. The two electrodes were simultaneously advanced in small discrete steps of ~0.1 mm and typical recording duration of ~20 s within the STN along the planned trajectory axis (recording durations were increased at sites of vocal emotional stimuli to ~180 s). A synchronized acquisition of the MER data and played emotional voices was performed. The emotional voices were randomized and a pseudo-random set (of actors and emotions) was played 1–3 times/trajectory in accordance with the patient's condition and preference. The STN entry and exit were discerned visually by the neurophysiologist as a sharp increase and decrease in the background activity, respectively. The STN boundaries were further confirmed and the dorsolateral oscillatory region was detected automatically using a custom method (Zaidel et al., 2009, 2010). Visual display of a typical trajectory data is presented at Figure 1. The root mean square (RMS) of the MER signal is normalized by its value at non-cellular area (white matter of the anterior internal capsule before the STN entry). The normalized RMS at the different recording depths is used to estimate the spiking (multi-unit) background activity and to define the MER depths at which the electrode intersects the dorsal STN entry and ventral STN exit (Figure 1, upper plot). Spectrograms (power spectral density as a function of the recording depth) facilitate the subdivision of the STN into a dorso-lateral oscillatory region and a ventro medial non-oscillatory region (Figure 1, lower plot). Typically, 80–100 MER sites were recorded for each hemisphere over 40 min. Of this time 10–15 min were usually dedicated to perform the current experiment. The neurosurgeon and the psychiatrist continuously evaluated the patient condition during the surgery. The experimental procedure was stopped if the patient expressed unwillingness to continue or if prolonging the surgery was deemed inadvisable.

### Neuronal data recording and analysis

In the operating room, 44 emotive voices were played for durations of 1–3 s with inter-voice random intervals of 2–4 s in up to three locations within the STN. Seventy-one data segments from 25 STNs of the 17 patients were available for analysis (Table 2).

**Table 2:** **Details of recording locations**.

**Patient**	**# Recording sites**			
	**Left**		**Right**	
	**Dorso-lateral**	**Ventro-medial**	**Dorso-lateral**	**Ventro-medial**
1	2	0	0	0
2	1	0	0	0
3	0	0	1	4
4	1	2	2	2
5	1	1	0	0
6	1	0	0	2
7	1	2	0	0
8	2	2	2	0
9	0	2	1	3
10	0	2	0	0
11	0	0	3	2
12	3	2	1	2
13	1	2	2	2
14	2	2	1	4
15	1	0	0	0
16	2	0	0	0
17	0	2	0	0
Total	18	19	13	21

At first, the raw analogue signal was rectified by the “absolute” operator to detect burst frequencies below the range of the operating room 250–6000 Hz band-pass filter (Zaidel et al., 2010). The rectified signal was smoothed with a digital eight-order low-pass Chebyshev Type I software filter with cutoff frequencies of 80 and 400 Hz. Then, the signals were down-sampled at 200 and 1000 Hz for root mean square (RMS) and spectral analysis, respectively. The above low-pass filter thresholds and down-sample rates were selected empirically such that the RMS best represents the average background activity (low pass filtering at 80 Hz and down sampling at 200 Hz) and to allow an accurate spectral analysis (low pass filtering at 400 Hz and down sampling at 1000 Hz).

The RMS is related to the variability of the MER signal in the local (<0.1 mm) area of the electrode. Intensive and burst activity that characterizes the STN is associated with a high variability and therefore large RMS values. The RMS was computed with a bin size of 500 ms and bin steps of 20 ms on the down sampled rectified MER data. Then, the RMS Z-score was computed to indicate how the MER energy changed below or above the mean RMS during vocal emotive stimulation. The Z-score function parameters (mean and SD) were estimated from the RMS values 1 s before the voice stimulation and smoothed with a Gaussian window (window size = 35 ms).

To estimate if there was a significant RMS response with respect to electrode location, the average Z-score was computed over a bin size of 2 s (400 samples) after stimulation and over all the 44 played voices. A ~5% average Z-score increase was observed at many sites within the right ventro-medial non-oscillatory region, but not in the left ventro-medial non-oscillatory region or left or right dorso-lateral oscillatory regions. Therefore 5% was used as a cut-off to decide whether an STN site was responsive to the emotional voices. This low “experimentally driven” threshold is to be expected when averaging the Z scores of 400 samples. Similar results were found with different thresholds in the range of 3–7% (data not shown).

The RMS estimates the total energy of the STN signal integrated over all frequencies. Spectral analysis was performed to study the effect of the emotive vocal stimulation on the different frequency bands of the STN spiking activity. Specifically, the power spectrum density (PSD) and event related de-synchronization (ERD) in resolution of 1/3 Hz and in the range of 3–250 Hz were computed. As a preparation step for computing the average power spectral density, the 1000 Hz down sampled rectified MER data was truncated into segments of 2000 ms with steps of 50 ms between segments. Then, the power spectral density of each segment was computed using Welch's method with a 1 s Hamming window and 50% overlap. The event related de-synchronization was computed by dividing the resulted power spectral density by the average power spectral density 1 s before stimulation for each frequency bin (0.33 Hz). Thus, the event related de-synchronization normalizes out high power spectral density values in the beta (12–30 Hz) and gamma (30–100 Hz) bands frequencies that characterize the dorso-lateral and ventro-medial regions, respectively. Such location related features may mask a possible temporal response to emotive stimuli. The event related de-synchronization therefore estimates the temporal changes in the frequency domain after emotive stimulation. Specifically, modulations were expected to be observed at the alpha (8–12 Hz) and low beta (12–20 Hz) bands (Buot et al., 2012). The neural modulation was also analyzed with respect to valence (positive, neutral or negative) of the emotive voice. The average (over 2 s) responses were compared and a *t*-test was performed to evaluate the significance of their differences.

Artifacts (e.g., large transients induced by patient movement or staff handling of the patient and electrical artifacts during stimulation) were detected by visual inspection of the raw data. PSD plots, and MERs associated with these artifacts were excluded (1 right and 3 left trajectories). Analysis was performed with custom programs written in Matlab (The MathWorks, Inc., Massachusetts, US).

## Results

**Figure 2:**
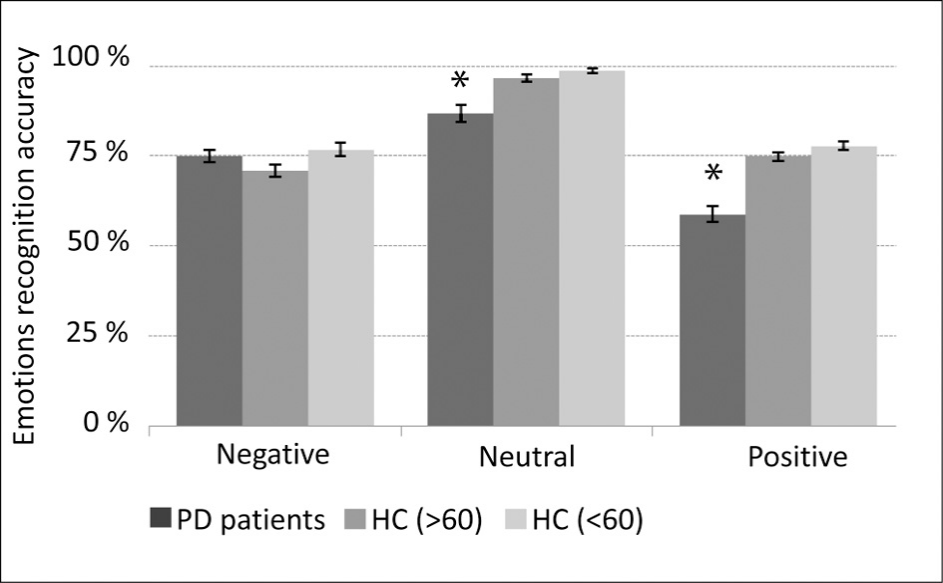
**Emotions recognition accuracy**. Medicated Parkinson's disease (PD) patients (*n* = 13) compared with three healthy control (HC) groups: (1) average age and gender (male/female) ratio matched (AGM) group (*n* = 12); (2) 60 years old or older (*n* = 14), and; (3) younger than 60 years old (*n* = 15). Error bars represent standard error of the mean (SEM). ^*^p < 0.05 (*t*-test) statistical significant difference between the PD patients and each of the tested healthy control groups.

PD patients “on” medication (~24 h before surgery) less accurately recognized (*p* < 0.05) voices expressing positive and neutral emotions in comparison to healthy age and gender matched subjects (Figure 2).

**Figure 3:**
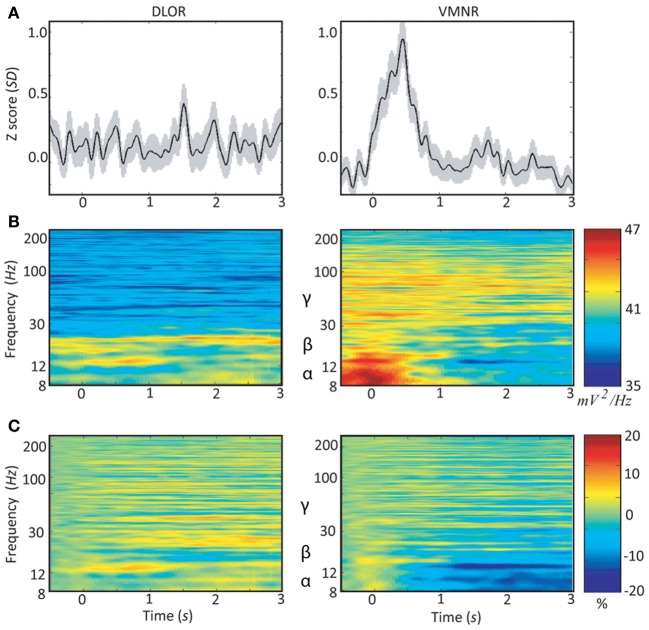
**Peri-stimulus time histograms (PSTH) and event related desynchronization (ERD) of two simultaneous microelectrodes recordings (MER) of the right subthalamic nucleus**. Data of patient #3 from the dorso-lateral oscillatory region (left column) and the ventro-medial non-oscillatory region (right column) during emotive stimulation with 44 voices (introduced at time = 0): **(A)** average Z-score of the root mean square (RMS). The light shadowing is the standard error of the mean (SEM). **(B)** Average power spectrum density (PSD); and **(C)** average event related desynchronization (ERD).

Neuronal responses to emotive stimuli in the ventro-medial non-oscillatory region of the right STN were far larger than the responses at the dorso-lateral oscillatory region. An example is presented in Figure 3. In this case one electrode was located in the ventro-medial non-oscillatory region of the right STN while the other was located 2 mm apart at the dorso-lateral oscillatory region of the same STN such that the spiking activity was recorded simultaneously from both regions and for the same vocally emotive stimuli. The Z score of the spiking activity increased in the ventro-medial non-oscillatory region after stimulation, but not in the dorso-lateral oscillatory region (Figure 3A right vs. left). Moreover, large decreases in the power spectrum density and event related desynchronization of alpha and low beta bands (8–12 and 12–20 Hz, respectively) were observed in the ventro-medial zone, but not in the dorso-lateral region (Figures 3B,C right vs. left). A comparison of the power spectral density values reveal that beta band (12–30 Hz) oscillatory activity was observed at the dorso-lateral oscillatory-region (Figure 3B left) before and after the presentation of the vocal stimuli (Time = 0). Gamma band (30–100 Hz) power spectral density values were larger at the ventro-medial non-oscillatory area in comparison to the dorso-lateral oscillatory-region (Figure 3B right vs. left).

**Figure 4:**
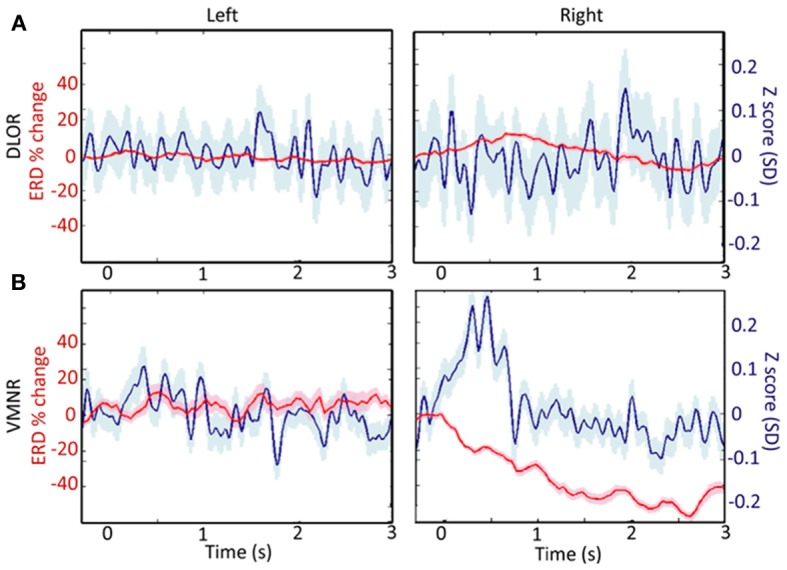
**A comparison of responses to emotive stimulation in different subthalamic nucleus (STN) regions. (A)** Left and right dorso-lateral oscillatory regions (DLOR, 14 STNs, 18 data segments, and 11 STNs, 13 data segments, respectively). **(B)** Left and right ventro-medial non-oscillatory regions (VMNR, 14 STNs, 19 data segments, and 11 STNs, 21 data segments, respectively). Significant increases in the background activity, and reduced oscillatory activity was observed in the VMNR of the right STN after vocal emotional stimuli **(B**, right**)**, but not in the left VMNR **(B**, left**)** or left or right STN DLOR **(A)**. Solid line represents the response mean, and shaded area represents the standard error of the mean (SEM).

**Figure 5:**
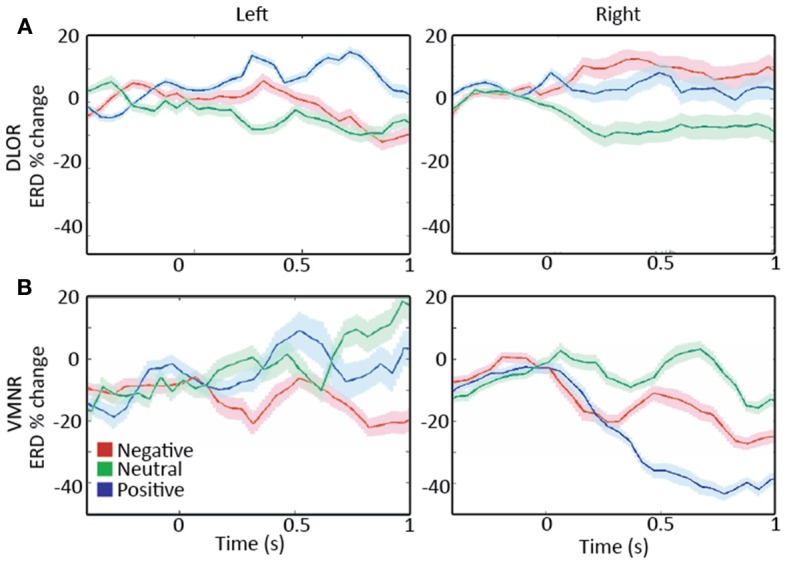
**A comparison of responses to emotive stimulation in subthalamic nucleus (STN) regions. (A)** Left and right dorso-lateral oscillatory regions (DLOR), 5 STNs, 7 data segments, and 3 STNs, 4 data segments, respectively. **(B)** Left and right ventro-medial non-oscillatory regions (VMNR, 5 STNs, 8 data segments, and 4 STNs, 10 data segments, respectively). A steep reduction in the oscillatory activity is present in the right STN VMNR for positive voices **(B**, right**)**, but not for neutral voices and less for negative voices. Such responses are not observed for emotive voice stimuli in the left VMNR **(B**, left**)** or left or right STN DLOR **(A)**. Color coding: Red, green, and blue responses to negative, neutral, and positive emotive voices, respectively. Solid lines represent the mean of the event related desynchronization change (ERD), and the surrounding shaded area represents the response standard error of the mean (SEM).

Analyzing the STN spiking activity of all 17 patients collectively (12 bilateral and 5 left unilateral DBS surgeries; 11 right and 14 left STNs after exclusion of noisy data, Tables 1, 2) supported these findings. The impact of emotional stimuli on the event related de-synchronization and the Z-score spiking activity was different in different STN domains (Figure 4). The right ventro-medial non-oscillatory of the STN was associated with large responses (reduction in event related de-synchronization and increased Z score spiking activity) to the vocally emotive stimuli (Figure 4B, right). The neuronal responses in the right ventro-medial non-oscillatory region were of larger magnitude (paired *t*-test *p* < 0.01) in comparison to the responses in the left ones (Figure 4B, left) and to the left and right dorso-lateral oscillatory-regions (Figure 4A, left and right). Moreover, larger responses were observed for positive, rather than negative or neutral, stimuli in the right STN ventro-medial non-oscillatory region (Figure 5; paired *t*-test *p* < 0.01).

Spatial analysis of the responses reveal that an average Z score increase of 5% or more (see Methods section for more information) was observed in 48% (10/21) of MER sites in the right ventro-medial non-oscillatory region, in comparison to only 11% (2/19) of sites in the left ventro-medial non-oscillatory region, and with 23% (3/13) and 22% (4/18) of the sites in the right and left dorso-lateral oscillatory-regions, respectively. Moreover, most of the responses in the right and left dorso-lateral oscillatory-regions (66% and 75%, respectively) are near its border with the ventro-medial non-oscillatory region and might reflect a fuzzy boundary between the different territories of the STN.

**Figure 6:**
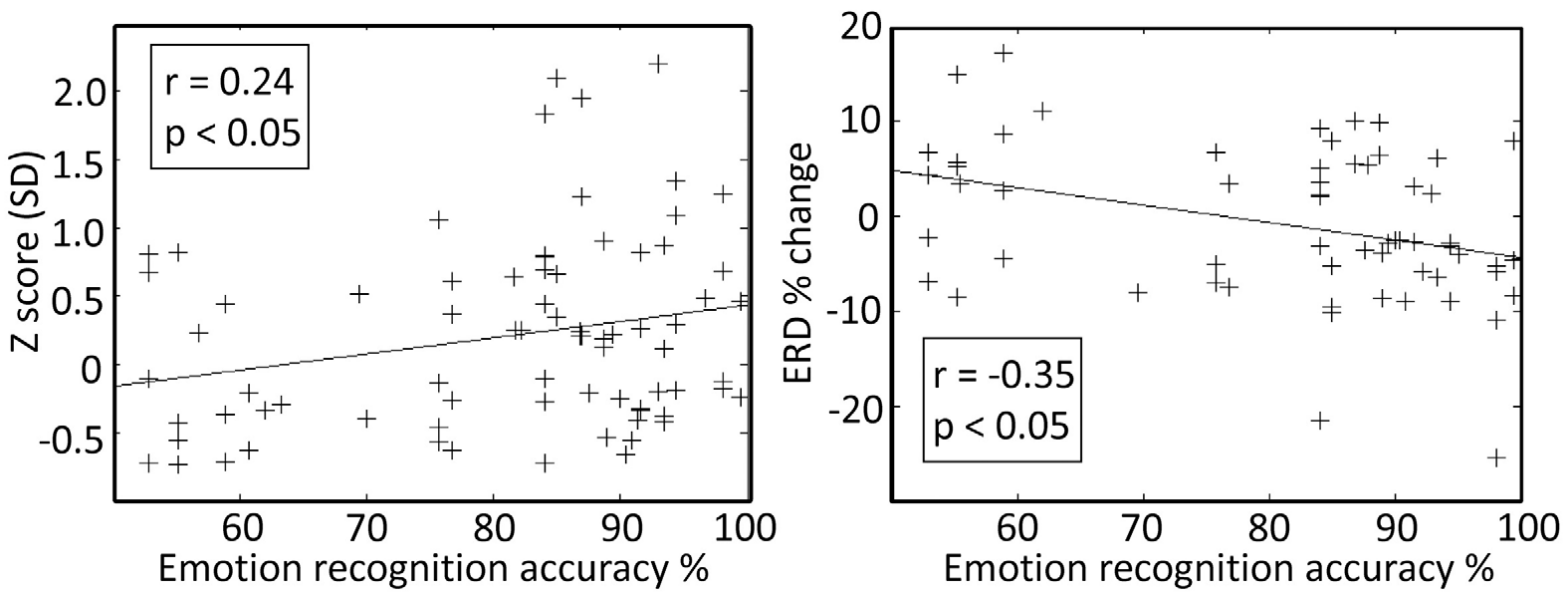
**Preoperative emotion recognition accuracy is related to the intraoperative responses at the right subthalamic nucleus (STN) ventro-medial non-oscillatory region (VMNR)**. Each “+” represents a response to a single voice presentation. Significant correlations were observed between the preoperative emotion recognition accuracy and changes in STN background activity (e.g., Z score), and changes in oscillatory activity [e.g., event related de-synchronization (ERD)].

Finally, intra-operative responses (7 right ventral STNs, 8 data segments) to vocally emotive stimuli were compared to the results of the preoperative MAV battery. Intraoperatively, patients were off medications, while preoperative testing was performed with patients on medications to ensure highest emotional recognition ability and to minimize the preoperative stress. Analysis of the intraoperative responses was carried only for emotive voices that the patient had reported preoperatively as reflecting increased arousal and non-neutral valence. Significant correlation (*p* < 0.05) was observed between preoperative emotion recognition accuracy and intraoperative spiking activity (Z score and event related de-synchronization) for the emotive voices (Figure 6; *r* = 0.24, and *r* = −0.35, respectively). Significant correlation was also observed between preoperative perceived arousal of the emotive voice and intraoperative modulation of the neural activity amplitude (Z-score, *r* = 0.25, *p* < 0.05).

## Discussion

These results provide electrophysiological evidence for lateralization of emotional brain function in the human basal ganglia. They also further support the concept of segregation of the STN into separate motor and non-motor regions and suggest that these regions may be differentiated by specific electrophysiological markers.

### STN functional organization

Early evidence for the somatotopic organization of the STN can be tracked back to non-human primate studies in the late 1940's (Mettler and Stern, 1962). Lesions at some STN areas of a Rhesus monkey have resulted with ballistic movements, while other areas were associated with a choreic type of movement, or were not associated with abnormal movements at all. Studies of the basal ganglia of behaving primates have revealed a somatotopic organization of movement related neurons (Delong et al., 1984; Wichmann et al., 1994; Nambu et al., 2002). Autoradiographic tracer studies have demonstrated that the ipsilateral STN receives a somato-topically organized projection from the pre-central motor cortex (Monakow et al., 1978). The remaining part of the nucleus was related with less intensive projections from the premotor and prefrontal areas. Nambu et al. (1996; Nambu, 2011) found that inputs from the primary motor cortex were allocated mostly within the lateral half of the STN (hyperdirect pathway) and a somatotopic organization was demonstrated for the orofacial, forelimb, and hindlimb subdomains. Haynes and Haber (2013) have recently observed topographically organized cortico-STN pathway in primates. They report that limbic areas project to the medial tip of the STN, straddling its border and extending into the lateral hypothalamus. Associative areas project to the medial half of the STN, and motor areas to the lateral half. Moreover, limbic projections terminated primarily rostrally and motor projections more caudally. An imaging study on human subjects suggests that the STN can be divided bilaterally into limbic, associative and motor regions occupying the anterior, mid and posterior portions of the nucleus respectively (Lambert et al., 2012).

Microelectrode recordings (MER) are often utilized in DBS surgeries to define the STN boundaries (Hutchison et al., 1998; Benazzouz et al., 2002; Castner et al., 2007) and facilitate the spatial mapping of its physiological subdomains. Rodriguez-Oroz et al. (2001) have studied STN MER of PD patients and reported that all neurons with sensorimotor responses were in the dorsolateral region of the STN. Abosch et al. (2002) also reported that movement related responses were observed more in the dorsal part of the STN in comparison to its ventral area. Further studies incorporating MER reported even more detailed somatotopic organization, and revealed that arm-related STN cells were located laterally and at the rostral and caudal poles, whereas leg-related cells were located medially and centrally (Rodriguez-Oroz et al., 2001; Theodosopoulos et al., 2003; Romanelli et al., 2004). Clinically, most effective DBS treatment is associated with stimulation of the dorsal STN region (Godinho et al., 2006; Weise et al., 2013). Neural activity of the anterior-medial area of the STN can be correlated with checking behavior in the STN of patients with OCD (Burbaud et al., 2013). Another recent study incorporating LFP signals from PD STN's, demonstrated that the ventral part of the STN encodes the emotional valence of stimuli independently of the motor context (Buot et al., 2012).

Our results support the subdivision of STN into dorsal-“motor” and ventral-“non-motor” regions. These findings may explain previous reports on emotional changes in PD patients undergoing DBS in the STN (Temel et al., 2006; Mallet et al., 2007; Witt et al., 2008). Finally, our findings provide supportive evidence for the limbic role of the right ventral STN (Péron et al., 2013) and its involvement in encoding of emotional prosody (Alba-Ferrara et al., 2011).

### Dopaminergic modulation of emotional processing in PD

It has been proposed that dopaminergic medication may reverse the bias of non-medicated PD patients for better learning from negative feedback and make them more sensitive to positive than negative outcomes (Frank et al., 2004; Bódi et al., 2009; Maia and Frank, 2011). Therefore, it might be expected that PD patients on medication would better recognize positive emotions. However, our results demonstrate a significant impairment in recognition of non-verbal vocal burst representing positive emotions, but not for negative ones in the medicated PD patients tested before their DBS procedures. The reported results of PD emotion-recognition studies are mixed (Kan et al., 2002; Assogna et al., 2010; Moustafa et al., 2013). The discrepancies between the studies may be related to the stimulation modality (e.g., visual vs. auditory; Kan et al., 2002), the type of dopaminergic therapy (e.g., Levodopa vs. dopamine agonists; Moustafa et al., 2013), the intensity of presented emotions (Assogna et al., 2010), and the testing conditions (i.e., high patient stress and anxiety at the day before surgery in our setup).

Previous studies utilizing macro-electrode LFP recordings in the STN of PD patients have demonstrated event related de-synchronization activity in the alpha and low beta frequencies (8–20 Hz) after emotive stimuli (Kühn et al., 2005; Brücke et al., 2007; Huebl et al., 2011; Buot et al., 2012). The largest modulation was observed after a visual presentation of pleasant stimuli in patients “on” dopamine medication. Bout et al. reported that largest modulation in “off” dopamine medication PD patients was caused by unpleasant stimuli (Buot et al., 2012). This observation adds important new evidence regarding the effect of dopaminergic drug therapy on emotional processing (Castrioto et al., 2013). The discrepancy between the reported LFP results and those in this study may be related to the differences in stimulation modality, intensity of presented emotions and the high patient stress and anxiety during surgery.

Another marked difference between our results and previous LFP studies is that no significant lateralization of the STN responses were observed in macro-electrode recordings. This discrepancy may be explained by the greater spatial resolution afforded by microelectrode recording of spiking activity in comparison to macro-electrode LFP recording. Furthermore, LFP's probably mainly reflect the synaptic input of the STN (Buzsáki et al., 2012) while spikes recorded with microelectrodes reflect the output of the STN. It may be that there is right/left symmetry in the synaptic inputs to the STN and still right/left *asymmetry* in their output (e.g., due to different excitability levels).

## Concluding remarks

The involvement of the basal ganglia in reinforcement learning has been extensively studied (Schultz et al., 1997; Hollerman and Schultz, 1998; Morris et al., 2004; Bayer and Glimcher, 2005; Joshua et al., 2010). Effective reinforcement learning depends on accurate recognition of the current state of the animal, including the emotional valence of different stimuli. Our results show that the largest activity modulation was associated with positive emotional stimuli and therefore the right ventral STN likely encodes emotional information that may be incorporated in reinforcement learning.

The results of this imply that DBS of the right ventral STN might be associated with more psychiatric side effects in PD patients in comparison to other STN regions. Therefore, the right ventral STN should be identified during the implantation of the DBS electrodes. We suggest that adjustment of STN DBS stimulation parameters should take into account emotional symptoms and could employ different strategies for the right and left STN to improve treatment outcome. Special caution is advised with right-sided stimulation in patients that are prone to or have developed psychiatric side effects.

Further studies may investigate the potential benefit of ventral STN DBS for primary psychiatric disorders such as depression or OCD. It is further suggested that these studies will examine the possibility that STN DBS for psychiatric indications might be right unilateral rather than bilateral. Finally, it is our hope that further studies will also explore the hypothesis that the left ventro-medial non-oscillatory region of the STN is related to other non-motor functions that are lateralized to the dominant hemisphere such as speech (Anzak et al., 2011).

### Conflict of interest statement

The authors declare that the research was conducted in the absence of any commercial or financial relationships that could be construed as a potential conflict of interest.
